# Critical Age-Friendly Research and Representational Ethics

**DOI:** 10.1093/geroni/igab046.1538

**Published:** 2021-12-17

**Authors:** Austin Oswald

**Affiliations:** CUNY Graduate Center, New York, New York, United States

## Abstract

As the efforts of the Global Age-friendly Cities and Communities movement mature and continue to grapple with society’s shifting dynamics, blind spots and knowledge gaps are exposed. This research applies critical discourse analysis to examine the evolution of Age-friendly NYC using an intersectional lens committed to an ethics of representation. Over 1,000 pages of public records were analyzed to trace the history of this movement in relation to age, race, sexuality, gender, ability, and class. Findings suggest that Age-friendly NYC is a global leader of the age-friendly movement, yet social identities are represented neither equally nor universally in its initiatives. Discussions of race, sexuality, and gender are subtle. They also overlook how these identities may intersect and shape the aging experience for differently positioned older adults. A comprehensive understanding of the aging experiences of those with multiple intersecting identities is needed to inform future age-friendly policies and programs.

